# Evaluation of age-dependent morphometrics of the meniscofemoral ligaments in reference to the posterior cruciate ligament in routine MRI

**DOI:** 10.1007/s00330-017-5128-x

**Published:** 2018-01-10

**Authors:** Sebastian Röhrich, Franz Kainberger, Lena Hirtler

**Affiliations:** 1 Department of Biomedical Imaging and Image Guided Therapy, Medical University of Vienna, Vienna, Austria; 2 Division of Anatomy, Centre for Anatomy and Cell Biology, Medical University of Vienna, Währingerstraße 13, 1090 Vienna, Austria

**Keywords:** Meniscofemoral ligaments, Posterior cruciate ligament, Routine MRI, Age-dependent changes, Morphometrics

## Abstract

**Objectives:**

To quantify the morphological correlation between the posterior cruciate ligament (PCL) and the meniscofemoral ligaments (MFLs), to propose normal ranges for different age populations, and to define guidelines for correct identification and differentiation of MFLs in routine MRI.

**Methods:**

Three hundred forty-two subjects were included retrospectively and subdivided into five age groups. Morphometrics of the PCL and the MFLs were measured on standard MRI in the sagittal, coronal, and axial planes. Student’s *t* test, Mann-Whitney *U* test, and ANOVA and Kruskal-Wallis tests with Bonferroni correction were used for comparison.

**Results:**

The MFLs did not vary significantly between sexes (*p* > 0.05) or in those older than 10 years (*p* > 0.05). Longitudinal MFL growth is completed before age 11 years, with cross-sectional area (CSA) increasing until age 20. The CSA of the PCL was significantly (*p* = 0.028) larger in knees without a pMFL (Mdn = 39.7 mm^2^) than with a pMFL (Mdn = 35.4 mm^2^). MFLs were more often detected on sagittal than coronal images.

**Conclusions:**

This study describes the morphometric relation between the PCL and the MFLs on routine MRI. When reporting imaging findings in preparation for arthroscopic knee surgery, evaluation of MFLs, first in the sagittal and then the coronal plane, will achieve the best results.

***Key Points*:**

*• The MFLs and the PCL have distinct morphological patterns throughout life.*

*• These patterns show intimate anatomical relationships and a potential biomechanical impact.*

*• Those patterns and relationships can be quantified with MRI.*

*• A correlation exists between age and morphometrics of the MFLs.*

*• Recommendations for correct identification of the MFLs are provided.*

## Introduction

**Fig. 1:**
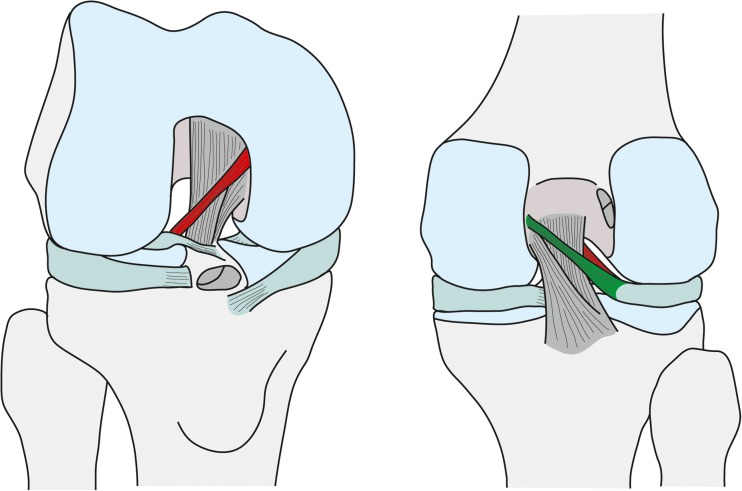
Anatomy of the meniscofemoral ligaments. The anterior meniscofemoral ligament (aMFL, red) is located anterior to the posterior cruciate ligament (PCL), and the posterior meniscofemoral ligament (pMFL, green) posterior to it

As scientific knowledge about the anterior (aMFL, ligament of Humphry) and posterior (pMFL, ligament of Wrisberg) meniscofemoral ligaments (MFLs, see Fig. 1) grows, a recognition of their relevance does as well. However, findings on parameters concerning the morphology of the MFLs differ [1–5], creating an ongoing opportunity for further clarification. One explanation for this documented variability may be found in the work of Woo et al. [6], who showed that the ligaments in the knee joint were significantly stronger in young adults than in the elderly. Age-related degeneration of the MFLs would involve not only a weakening of the MFL structure, but also the possible eventual disappearance of the MFLs, as studies have also suggested [7, 8].

The clinical significance of the MFLs has been shown in several scientific reports. A recent study [9] describes a correlation between the trauma mechanism in anterior cruciate ligament rupture and posterior lateral meniscal root tears with concomitant MFL injury, and proposes a revised treatment strategy based on the extent of MFL involvement. Special considerations arise in cases of posterior cruciate ligament (PCL) injuries, as the MFLs can remain intact while the PCL ruptures [1, 10], thus functioning as auxiliaries to the weakened PCL and stabilising against posterior drawer. Accordingly, synergisms between the MFLs and the PCL have been described on many levels [2, 10–16]. In addition, in the development of knee osteoarthritis (OA) [17], damaged MFLs may constitute a contributory risk factor due to the increase in femorotibial contact pressure [11, 18–21].

**Fig. 2:**
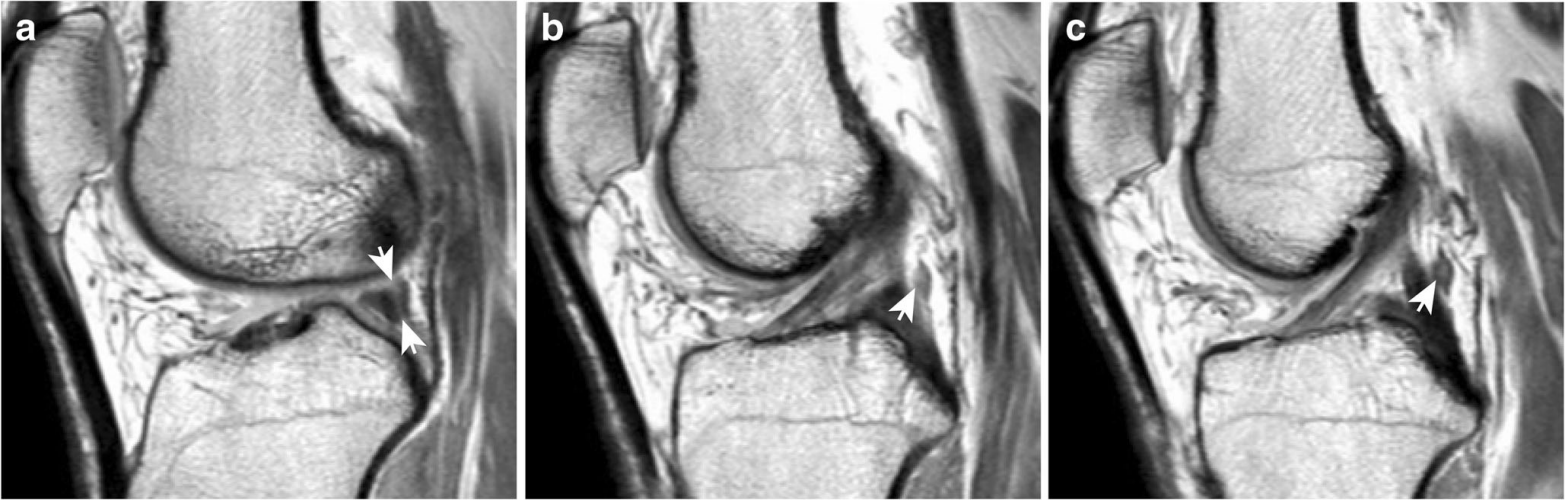
Three consecutive sagittal images of the right knee of a 52-year-old man from lateral to medial. (A) A hyperintense “lesion” (arrows) can be seen just posterior to the posterior horn of the lateral meniscus. This appearance corresponds to the pMFL detaching from the meniscus. (B) The pMFL (arrow) takes a rounder shape as it departs farther from the lateral meniscus and the gap in between grows in width. (C) The pMFL (arrow) can be seen as an autonomous structure posterior to the PCL

Whereas in the past, correct identification of the MFLs has helped to differentiate a physiological structure from lateral meniscal tears in magnetic resonance imaging (MRI) [22–24] (see Fig. 2), the correct identification of MFL injury may now be required by the orthopaedic surgeon as part of the pre-surgical diagnostics [21]. However, standardised guidelines for MRI identification are still lacking.

The aims of this study were (1) to quantify the morphological relationship between the PCL and the MFLs, (2) to propose normal ranges for different age populations, and (3) to define guidelines for the correct identification and differentiation of the MFLs in routine MRI.

## Materials and methods

This cross-sectional study was carried out retrospectively. After ethical approval was obtained, we evaluated all knee MRIs of regular patients from our clinic (*n* = 1880), performed on the same 3-Tesla MRI scanner during the period from 2007 to 2012.

### Subjects

Subjects were excluded if they had or showed signs of any of the following: arthroscopy or intraarticular surgery; rupture; lesion or degeneration of the cruciate ligaments and the lateral or medial meniscus; capsular damage; arthritis; infection or tumour of the knee; moderate or severe knee OA (Kellgren and Lawrence grade 3/4 criteria for projection radiographs were applied to MRI accordingly); reported varus or valgus deformity (> 15°); or poor image quality.

The remaining subjects (*n* = 342) comprised 55.3% female (*n* = 189, mean age 32 years, range 1–77 years) and 44.7% male patients (*n* = 153, mean age 32 years, range 2–73 years).

**Fig. 3:**
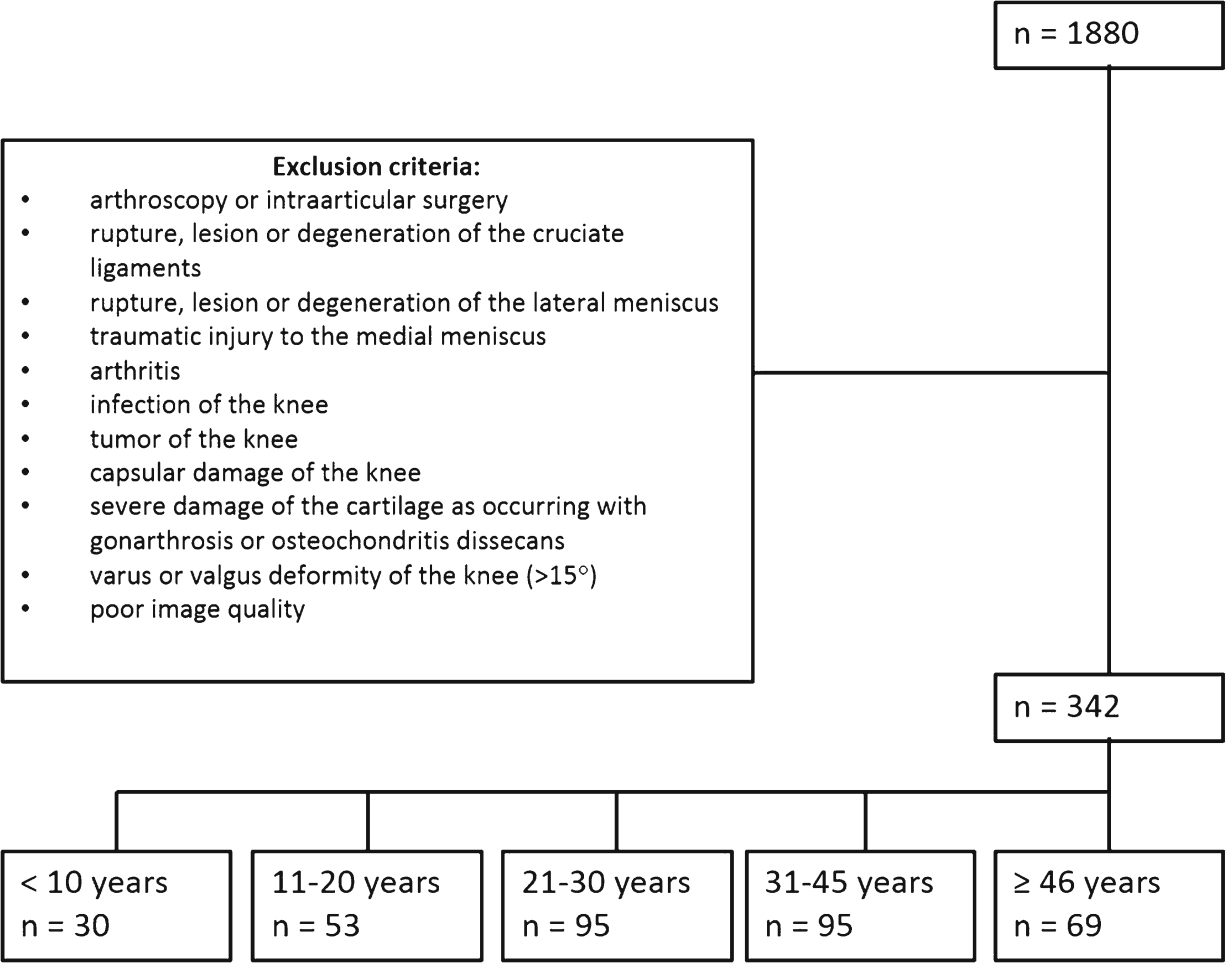
Diagram of exclusion criteria applied to the population and subdivision into age groups

After the selection process, the subjects were subdivided into five age groups according to their age at the time of the MRI examination (see Fig. 3). For the lower age groups, the definition of the ranges was oriented toward the complete ossification of the femoral and tibial bone [25–28], whereas for the upper age groups the range was defined based on degenerative changes to the musculoskeletal system [29–31].

Only baseline exams were used, and in the case of bilateral images, only one side was examined.

### Methods

All images were acquired on a 3-Tesla MRI scanner (Philips Achieva® 3.0T; Philips Healthcare, Best, The Netherlands) with a standard knee coil (Philips SENSE® Knee, 8 elements; Philips Healthcare, Best, The Netherlands). All subjects were scanned using the same protocol: sagittal T1-weighted images; sagittal T2-weighted images with fat saturation; and sagittal, coronal, and axial proton density-weighted images with fat saturation. Measurements were obtained digitally with commercially available software (icoView®; ITH icoserve, Innsbruck, Austria).

### Morphometric analysis

Analysis of the MR images was performed by two investigators with at least 3 years of experience in musculoskeletal MRI interpretation. Each investigator was blinded to the other’s findings to enable inter-rater evaluation. In case of differences regarding the presence of an MFL, a joint decision was made. For continuous variables, the arithmetic mean of the investigators’ measurements was taken as the final value.

**Fig. 4:**
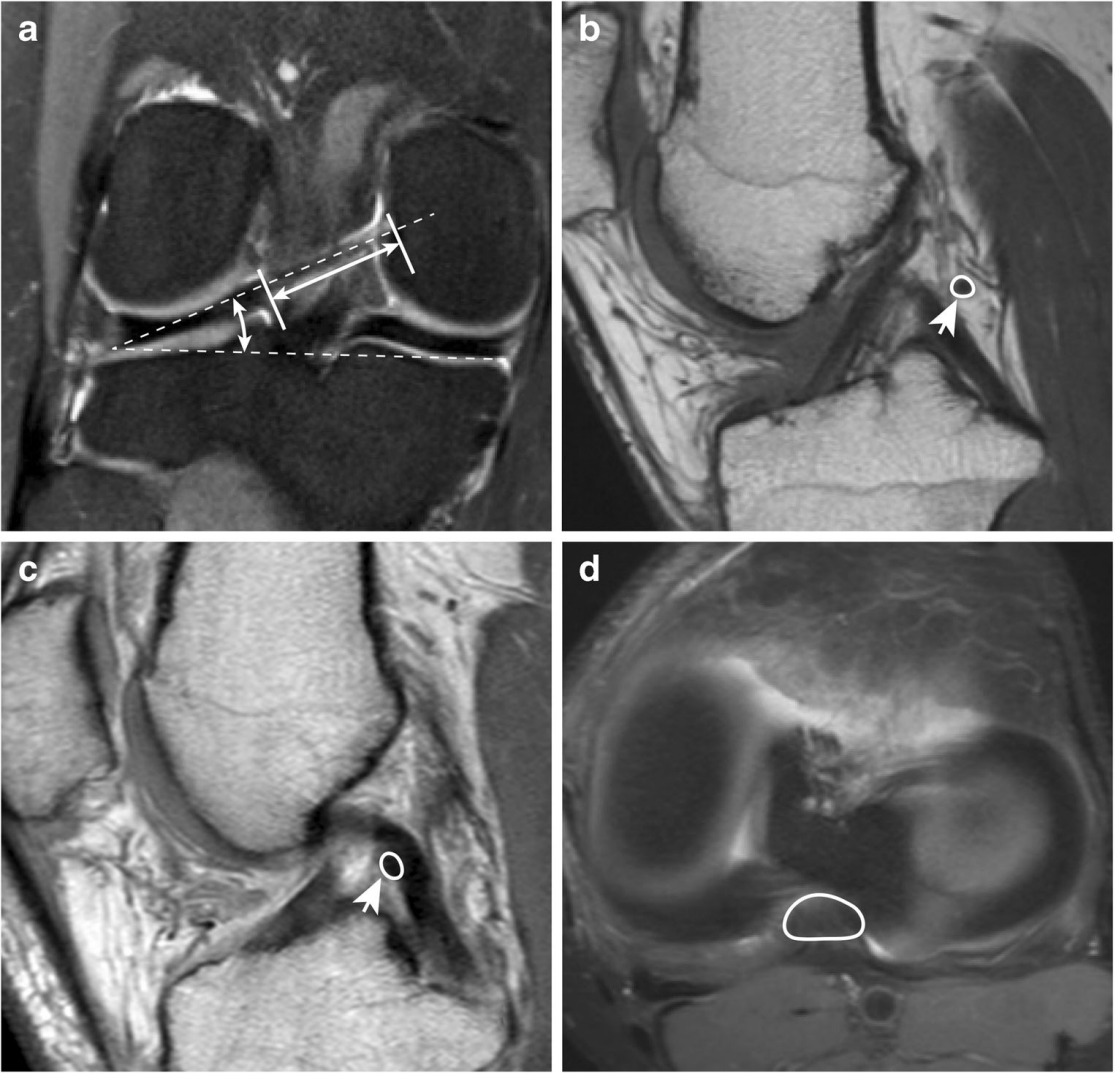
(A) On the coronal image of the right knee of a 29-year-old woman, the pMFL can be seen as a straight-lined hypointense band posterior to the PCL (arrow), allowing measurement of the length of the ligament. The angle between the longitudinal axis of the pMFL and the line connecting the distal margins of the medial and lateral femoral condyles in the coronal plane is referred to as the running angle of the pMFL [3]. (B) On the sagittal image of a 21-year-old man, the CSA of the pMFL is measured, seen as a hypointense dot posterior to the PCL (arrow). (C) On the sagittal image of a 25-year-old woman, the aMFL CSA is measured, seen as a hypointense dot anterior to the PCL (arrow). (D) In an axial image from the same subject as in B, the CSA of the PCL is measured at the height of the menisci

The following variables were measured: presence or absence and length and running angle of the MFLs in the coronal plane; cross-sectional area (CSA) of the MFLs at the midpoint of length in the sagittal plane; CSA of the PCL on the level of the joint line in the axial plane (exemplary measurements can be seen in Fig. 4); and detectability of the MFLs according to the MRI planes.

#### Prevalence

The presence of the MFLs was studied on both coronal and sagittal images. If an MFL was visible in at least one plane, it was classified as present. Close attention was paid to distinguish the oblique bundle of the PCL [7, 10] from the pMFL.

#### *Length* (see Fig. 4A)

After an MFL had been identified, the one coronal slice was chosen in which the course of the MFL from its meniscal origin to its femoral insertion was best depicted. The length was measured from the point of separation at the meniscal insertion to the lateral wall of the medial femoral condyle. To normalise the length of the MFLs to a reference, the notch width index (NWI) was calculated as the ratio of the intercondylar notch (ICN) width and the bicondylar width at the height of the popliteal groove [32].

*The running angle of the pMFL* (see Fig. 4A) was measured using the method described by Cho et al. [3].

*CSA:* The CSA of the MFLs was measured at the midpoint of the length of the MFL in the sagittal plane (see Fig. 4B and C). The CSA of the PCL was obtained in the axial plane at the level of the joint line (see Fig. 4D).

#### MFL detectability

For each MFL, each investigator documented the optimal plane for identification of the respective MFL.

### Statistical analysis

The statistical analysis was performed with IBM SPSS Statistics 21.0 software (IBM Corp., Armonk, NY, USA). A *p* value of < 0.05 was considered statistically significant. For metric variables, differences between male and female subjects were tested for significance with Student’s *t* test or the Mann-Whitney *U* test in the case of non-normally distributed variables (CSA of the MFLs). For analysis of categorical variables, the chi-square test was applied. An analysis of variance (ANOVA) was used to test for differences between age groups using Tukey’s post hoc test and the Games-Howell procedure. An alternative to an ANOVA in the event of non-normally distributed variables was the Kruskal-Wallis test, followed by Mann-Whitney tests with a Bonferroni correction, which led to a 0.0125 level of significance in all cases where it was applied (CSA of the aMFL and the pMFL compared between age groups). The results of the two independent investigators were compared using Cohen’s kappa coefficient [33] and the intraclass correlation coefficient (Table 2). We conducted a power analysis with an estimated level of significance of 5% (*z* score 1.65), power of 80% (*z* score 0.8416), expected effect size of 0.121 (relative difference in CSA of PCL according to the presence or absence of the pMFL) and standard deviation in the population of 0.297 (relative standard deviation of the CSA of the PCL). The calculated necessary sample size for detecting a difference was 74.8, compared to our study population of 342.

## Results

MFLs were identified in 324 of 342 subjects. An overview of descriptive measurements is listed in Table 1.

**Table 1:** Descriptive overview of the age groups, prevalence, and morphometrics of the MFLs and PCL

Age group (years)		All	≤ 10	11–20	21–30	31–45	≥ 46
Subjects	Total	342	30	95	95	53	69
	Male	153	18	42	43	20	30
	Female	189	12	53	52	33	39
Prevalence	At least one MFL (% of total subjects)	323 (94%)	28 (93%)	47 (89%)	90 (95%)	90 (95%)	68 (99%)
	Total aMFL (% of total subjects)	241 (71%)	22 (73%)	36 (68%)	67 (71%)	62 (65%)	54 (78%)
	Total pMFL (% of total subjects)	244 (71%)	25 (83%)	36 (68%)	66 (70%)	72 (76%)	45 (65%)
	Both MFLs (% of total subjects)	162 (47%)	19 (63%)	25 (47%)	43 (45%)	44 (46%)	31 (45%)
Length	aMFL (mean ± SD [mm])	22 ± 3	20 ± 3	22 ± 3	22 ± 3	22 ± 3	23 ± 3
	pMFL (mean ± SD [mm])	28 ± 4	23 ± 4	27 ± 4	28 ± 4	28 ± 4	29 ± 4
Running angle	pMFL (mean ± SD)	31 ± 6°	25 ± 6°	31 ± 6°	32 ± 6°	32 ± 5°	30 ± 6°
CSA	aMFL (mean ± SD [mm^2^])	2.2 ± 1.7	1.6 ± 1.3	1.8 ± 1.4	2.7 ± 2	2.4 ± 1.9	2.7 ± 1.9
	pMFL (mean ± SD [mm^2^])	3.3 ± 2.6	2 ± 1.6	3.4 ± 2.8	3.4 ± 2.7	3.6 ± 3.2	4.1 ± 2.8
	PCL (mean ± SD [mm^2^])	35.3 ± 10.5	24.6 ± 8.6	35.7 ± 10.6	38.9 ± 11.9	36.3 ± 9.1	40.9 ± 12.1
Detectability	aMFL sagittal (% of total aMFL)	238 (99%)	22 (100%)	35 (97%)	67 (100%)	60 (97%)	54 (100%)
	aMFL coronal (% of total aMFL)	190 (79%)	17 (77%)	25 (69%)	47 (70%)	56 (90%)	45 (83%)
	pMFL sagittal (% of total pMFL)	239 (98%)	25 (100%)	34 (94%)	66 (100%)	71 (99%)	43 (96%)
	pMFL coronal (% of total pMFL)	219 (90%)	23 (92%)	30 (83%)	56 (85%)	67 (93%)	43 (96%)

SD = standard deviation

**Table 2:** Kappa values and ICC of measured parameters

	Measured parameter		CI
Kappa values	Presence of the aMFL in the coronal plane	0.62	[0.32, 0.85]
	Presence of the pMFL in the coronal plane	0.71	[0.4, 0.87]
	Presence of the aMFL considering all of the available planes	0.84	[0.76, 0.96]
	Presence of the pMFL considering all of the available planes	0.96	[0.89, 0.98]
ICC	aMFL length	0.61	[0.39, 0.92]
	aMFL CSA	0.74	[0.41, 0.9]
	pMFL length	0.96	[0.9, 0.98]
	pMFL CSA	0.86	[0.7, 0.93]
	PCL CSA	0.8	[0.45, 0.9]

ICC = intraclass correlation coefficient, CSA = cross-sectional area, CI = 95% confidence interval

Kappa values according to Altman [34]: 0.81–1.00, very good; 0.61–0.80, good; 0.41–0.60, moderate; 0.21–0.40, fair; < 0.20, poor agreement

ICC values according to Cicchetti [35]: 0.75–1.00, excellent; 0.60–0.74, good; 0.40–0.59, fair; < 0.40, poor agreement

**Table 3:** Comparison of the prevalence of MFLs among studies

Author	Number of subjects/samples	At least one MFL	aMFL	pMFL	Both MFLs
Evaluation by MRI					
This study	342	323 (94.4%)	241 (70.5%)	244 (71.3%)	162 (47.4%)
Bintoudi et al. [8]	500	462 (92.4%)	59 (11.8%)	322 (64.4%)	81 (16.2%)
de Abreu et al. [22]	49	49 (100%)	27 (55.1%)	46 (93.9%)	22 (44.9%)
Lee et al. [36]	138	114 (82.6%)	6 (4.3%)	110 (79.7%)	2 (1.4%)
Nagasaki et al. [37]	38	32 (84.2%)	14 (36.8%)	27 (71.1%)	10 (26.3%)
Evaluation by arthroscopy					
Gupte et al. [38]	68	64 (94.1%)	60 (88.2%)	10 (14.7%)	6 (8.8%)
Niess et al. [39]	122	117 (95.9%)	71 (58.2%)	100 (82%)	52 (42.6%)
Evaluation by dissection					
Brantigan and Voshell [40]	50	50 (100%)	23 (46%)	33 (66%)	3 (6%)
Cho et al. [3]	28	25 (89.3%)	0 (0%)	25 (89.3%)	0 (0%)
de Abreu et al. [22]	10	10 (100%)	5 (50%)	7 (70%)	5 (50%)
Harner et al. [41]	8	8 (100%)	4 (50%)	6 (75%)	2 (25%)
Heller and Langman [4]	140	99 (70.7%)	50 (35.7%)	49 (35%)	8 (5.7%)
Kusayama et al. [2]	26	26 (100%)	18 (69.2%)	20 (76.9%)	12 (46.2%)
Nagasaki et al. [37]	30	30 (100%)	5 (16.7%)	30 (100%)	5 (16.7%)
Poynton et al. [42]	42	42 (100%)	35 (83.3%)	38 (90.5%)	27 (64.3%)
Wan and Felle [43]	60	60 (100%)	20 (33.3%)	56 (93.3%)	14 (23.3%)
Yamamoto and Hirohata [44]	100	100 (100%)	76 (76%)	73 (73%)	49 (49%)
Total	1409	1288 (91.4%)	473 (33.6%)	952 (67.6%)	298 (21.1%)

### Prevalence

There was no significant association between gender and the presence of the MFLs, nor was there a significant difference between the age groups.

### Length

Since the MFLs could not be depicted in the coronal plane in every subject, the length was measured only in those where the entire extent of the MFL was seen. Therefore, the length of the aMFL was measured in 190 cases and the pMFL in 219 cases.

On average, both the aMFL (*p* < 0.0001) and the pMFL (*p* < 0.0001) were significantly longer in male than in female subjects; however, there was no significant difference when length was normalised to the notch width index (NWI) (*p* > 0.05).

There was a significant effect of age group with regard to the length of the aMFL, (*p* < 0.001). The group ≤ 10 years of age had significantly shorter aMFLs than the groups aged 21–30 (*p* < 0.05), 31–45 (*p* < 0.05), and ≥ 46 years (*p* < 0.0001). No significant differences were found between the groups aged ≤ 10 years and 11–20 years, or between any of the other groups (all *p*s > 0.05).

There was also a significant effect of age group with regard to the length of the pMFL (*p* < 0.0001). The group aged ≤ 10 years had significantly shorter pMFLs than every other group: 11–20 years (*p* < 0.01), 21–30 years (*p* < 0.0001), 31–45 years (*p* < 0.0001), and ≥ 46 years (*p* < 0.0001). However, there were no significant differences between any other groups (all *p*s > 0.05).

### Running angle of the pMFL

On average, the running angle of the pMFL was larger in female (31.1 ± 5.6°) than in male patients (30.1 ± 6.1°); however, this difference was not significant. There was a significant effect of age on the running angle of the pMFL (*p* < 0.0001). The Tukey-honest significant difference (HSD) post-hoc test revealed that the running angles in the group aged ≤ 10 years were significantly smaller than those in all other groups (*p* < 0.01). There were no significant differences between any other groups.

### CSA

**Fig. 5:**
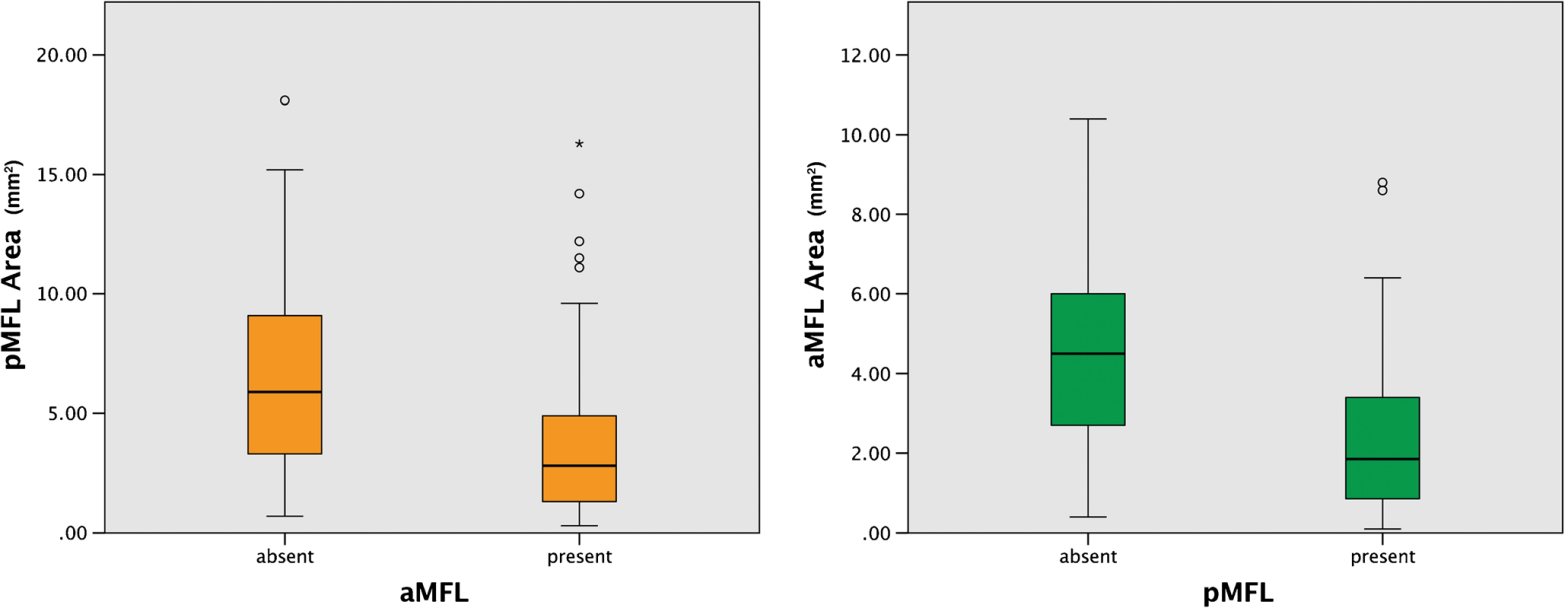
CSA of the pMFL in knees with a single aMFL or both MFLs (left image). CSA of the aMFL in knees with a single pMFL or both MFLs (right image)

Looking at all knees, the CSA of the pMFL was on average 2.97 times as large as that of the aMFL (range 0.08–22.9). Single aMFLs (Mdn = 4.5 mm^2^) had a significantly larger CSA than aMFLs in knees with both MFLs present (Mdn = 1.8 mm^2^, *p* < 0.0001; see Fig. 5). Single pMFLs (Mdn = 5.9 mm^2^) had a significantly larger CSA than pMFLs in knees with both MFLs (Mdn = 2.8 mm^2^*p* < 0.0001; see Fig. 5).

On average, the CSA of the aMFL was 9% of the CSA of the PCL (range < 1 to 35%), whereas the CSA of the pMFL was 13% of that of the PCL (range 1–43%). In knees with both MFLs present, their combined CSA was on average 17% of the CSA of the PCL (range 2–52%).

The Bonferroni correction was used to compare the CSAs of the aMFL and pMFL between age groups, resulting in a level of significance of 0.0125.

The CSA of the aMFL was significantly affected depending on the age group of the subject (*p* < 0.0001). There was no difference in aMFL CSA between groups aged 11–20 years (*r* = −0.25) and ≤ 10 years, whereas it was significantly larger in the group aged 21–30 years than in those aged ≤ 10 years (*p* < 0.0125).

No difference in aMFL CSA was found for either the 21–30-year (*r* = −0.01) or 31–45-year (*r* = −0.09) age groups compared to the oldest group, aged ≥ 46 years (all *p*s > 0.0125).

The CSA of the pMFL was significantly affected depending on the age group of the subject, (*p* < 0.01). No difference was found between the groups aged 11–20 years (*r* = −0.26) and ≤ 10 years (*p* > 0.0125). However, the CSA of the aMFL was significantly larger in the group aged 21–30 years than that aged ≤ 10 years (*r* = −0.29, *p* < 0.0125).

No difference in pMFL CSA was noted in the groups aged 21–30 years (*r* = −0.03) or 31–45 years (*r* = −0.02) compared to the oldest group, aged ≥ 46 years (all *p*s > 0.0125).

Further, there was no significant difference in the CSA of either the aMFL (*p* > 0.05) or pMFL (*p* > 0.05) between male and female patients.

### MFLs in relation to the PCL

**Fig. 6:**
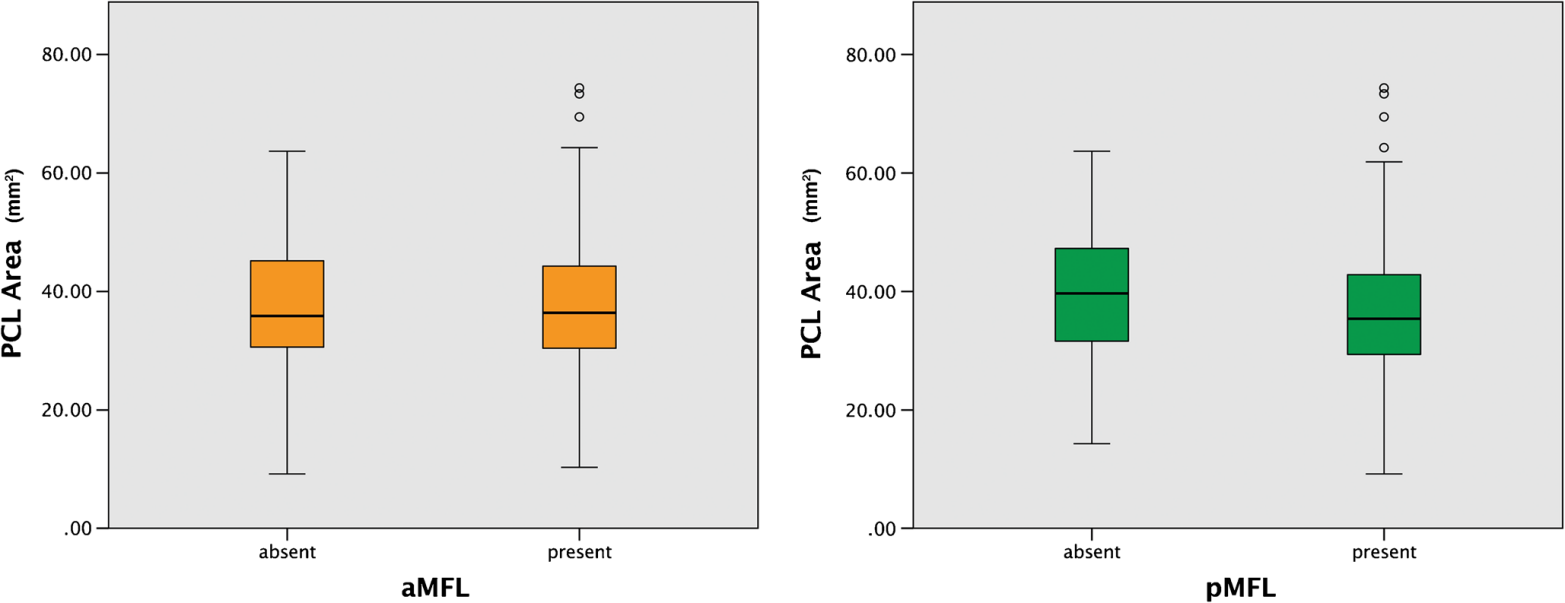
CSA of the PCL in knees with a present or absent aMFL (non-significant, left image). CSA of the PCL in knees with a present or missing pMFL (significant, right image)

The CSA of the PCL was nearly the same in knees with an aMFL present (Mdn = 36.4 mm^2^) as in knees with an absent aMFL (Mdn = 35.9 mm^2^). Thus, no significant difference was observed (*p* > 0.05: see Fig. 6).

Taken together, the CSA of the PCL was larger in knees with an absent pMFL (Mdn = 39.7 mm^2^) than with a pMFL present (Mdn = 35.4 mm^2^). This difference was significant (*p* < 0.05; see Fig. 6).

The presence (Mdn = 35.10 mm^2^) or absence (Mdn = 37.60 mm^2^) of both MFLs did not influence the CSA of the PCL (*p* > 0.05).

### MFL detectability in routine MRI

The detectability of the MFLs in the sagittal and coronal planes is shown in Table 1. In general, more MFLs were detected on sagittal images than on coronal images.

### Inter-rater agreement of measurements

Kappa values ranged from 0.62 to 0.96, indicating good to very good inter-observer agreement. ICC values ranged from 0.61 to 0.96, indicating good to excellent inter-observer agreement.

## Discussion

The results of this study are the first to show a clear morphological relationship between the PCL and the MFLs, as well as between single MFLs, to support the previously reported functional connection of ligaments in the knee over the life span [6]. We were able to identify the complex age-related changes in those structures among different age groups. Further, this paper establishes normal ranges for these ligaments from childhood to old age. The information provided, in combination with our proposed guidelines, will assist in the correct identification and differentiation of the MFLs in routine radiological practice.

At least one MFL was present in 94.4% of cases, which is similar to the findings of other authors (see Table 3). Contrary to some studies in which the pMFL was found to be present in nearly every case [22, 37, 43], the present study shows the pMFL to be present in 71.3% of cases. This difference is most likely based on the methodology used (dissection vs. MRI). It is possible that with the use of MRI evaluation, smaller MFLs are overlooked due to gap size or partial volume effects. This further underlines the importance of comprehensive anatomical knowledge of the region surrounding the posterolateral meniscal root and the ICN of the knee.

The pMFL clearly influences the morphology of the PCL. In our study, the PCL-CSA was significantly larger in knees where the pMFL was missing than in knees with a pMFL present. This larger CSA likely reflects an increase in fibres, as degenerative alterations of the PCL have not been observed in MRI studies before or during measurement. The pMFL is considered the larger of the two MFLs [3–5, 37] and supports the posteromedial bundle of the PCL synergistically [15]. Since the loading capacity of the posteromedial bundle reaches that of the anterolateral bundles only partly [14, 45], the PCL may have to compensate for a missing pMFL by being thicker, and therefore stronger, whereas in the case of an absent aMFL, the greater reserve capacity of the anterolateral bundle suffices.

The present study further shows that single MFLs have a significantly larger CSA than the same MFL in knees with both MFLs present. Thus, a single, strong MFL may be able to assume the role of both MFLs. The mean CSA of the MFLs in this study was smaller than that in most previously published data. However, the CSA of the pMFL in the present study was on average three times that of the aMFL, a ratio similar to those reported by other authors [3–5, 37, 46]. Nonetheless, there are huge discrepancies among findings in the literature [2, 5, 37]. One contributing factor is the inhomogeneity in the thickness of the MFLs along the axis of their length. The current study addressed this issue of comparability by choosing a measuring point at the midpoint of the length, similar to other studies [37, 42, 46, 47]. This approach seems to achieve more viable metrics for the CSA, because the variances have been mainly located at the meniscal origin [37, 39, 42] and the proximal insertion [3, 10]. Based on the morphological appearance, our study suggests the need for a larger single MFL to maintain physiological conditions in knees with only one MFL present.

There is no literature about the age correlation with MFL CSA, but several authors have noted a possible degeneration of the MFLs with age [7, 8] or have reported diminishing tensile strength in elderly populations [6], which may be explained by a thinning of the ligaments.

In this study, we found no correlation between age and the prevalence of the MFLs in healthy subjects. The CSA of the MFLs, however, increased in subjects until the age of 21 years, indicating ongoing growth during adolescence.

In accordance with other authors, we found no gender-related difference in the prevalence [4, 39] or the CSA [46, 47] of the MFLs. Further, a difference in length between male and female subjects became non-significant after normalisation with the ICN width.

With regard to the length of the MFLs, the only significant difference was in the group ≤ 10 years of age. Since the length of the MFLs depicts the distance between the lateral meniscus and the medial femoral condyle, it is plausible that MFL length remains constant after bone growth of the distal femur has finished. Therefore, while MFL growth in length is complete by the age of 11 years, the CSA continues to increase up to the age of 21 years.

The findings regarding the mean running angle of the pMFLs across age groups (30.7 ± 5.8°) were consistent with those reported in previous studies [3]. In addition, our study showed that the running angle of the pMFL was significantly smaller in children ≤ 10 years of age (25.2 ± 6.1°, *p* < 0.01). Similarly, an increase in the angulation of the ACL and the PCL in the knee joint prior to skeletal maturity was suggested to be the result of elongation of the intercondylar facet of the femoral condyles [48]. Due to the femoral insertion of the pMFL, this also offers a plausible explanation for the increase in the running angle.

For reliable identification of the MFLs on MRI, information from different, readily available slices should be combined and should always include the sagittal plane. Detectability in a single plane showed relevant variability, as seen in Table 1. The investigator should also keep in mind that the running angle changes during adolescence, and thus caution should be exercised to avoid mistaking the MFLs for meniscal structures.

In total, three specific conditions have been described in the literature in which an investigation of the state of the MFLs might provide relevant information: first, to support treatment decisions in cases of posterior lateral meniscal root tears [9, 21, 49–51]; second, in conjunction with ACL injury due to the correlation with concomitant injuries of the posterior horn of the lateral meniscus [9, 52–54]; and third, as a contributing factor to tibiofemoral contact pressure after rupture [21, 49, 51, 54–56], which in turn increases the risk for development of OA [19]. For an exact diagnosis, a thorough understanding of possible anatomical deviations is imperative.

The limitations of this study are its retrospective design and the evaluation of routine patient data. However, this shows that investigation of the presence or absence of MFLs is possible in routine clinical imaging. Due to the complex nature of various insertion types among the MFLs, we limited the CSA analysis to the midpoint of the MFL length, although estimations of parts closer to the femur and meniscus may yield additional information about possible correlations between the MFLs and structures of the knee. Also, due to the cross-sectional study design, there is no information available regarding longitudinal changes. A strength of this study is its large sample size. With 342 subjects, it surpasses most other MRI surveys of MFLs, as well as most of those using other research modalities. The focus on age groups allows for detailed insight into the morphometrics of the MFLs during different phases of life, especially in children. However, subjects older than 60 years who met the inclusion criteria were scarce, possibly due to the high incidence of knee pathologies in elderly populations. Thus, inclusion criteria may need to be revised in future studies, with the inclusion of groups older than 60 years. A comparison of healthy subjects to patients with knee OA may lead to a better understanding of the role of the MFLs in disease development.

In conclusion, we showed a clear morphological relationship between the PCL and the MFLs that will benefit future investigations. Due to the intricate morphological relationships of those structures and the functional implications in knee mechanics, the morphometric description of the MFLs may facilitate preoperative diagnostics in PCL, ACL, and meniscal surgery. A two-plane diagnostic approach, along with a thorough understanding of the age-related changes in MFL anatomy, is necessary for adequate image interpretation.

## Abbreviations

MFL: Meniscofemoral ligament
aMFL: Anterior meniscofemoral ligament
pMFL: Posterior meniscofemoral ligament
MRI: Magnetic resonance imaging
NWI: Notch width index
ICN: Intercondylar notch
ACL: Anterior cruciate ligament
PCL: Posterior cruciate ligament
CSA: Cross-sectional area
